# Older People and Social Connectedness: How Place and Activities Keep People Engaged

**DOI:** 10.1155/2012/139523

**Published:** 2012-01-04

**Authors:** Irene H. Yen, Janet K. Shim, Airin D. Martinez, Judith C. Barker

**Affiliations:** ^1^Department of Medicine, University of California, San Francisco, 3333 California Street, Suite 335, San Francisco, CA 94118, USA; ^2^Department of Social and Behavioral Sciences, University of California, San Francisco, 3333 California Street, Suite 455, San Francisco, CA 94143-0612, USA; ^3^Community Track Program, Department of Health, Johns Hopkins Bloomberg School of Public Health, Behavior and Society, 624 N. Broadway, HH 753, Baltimore, MD 21205, USA; ^4^Department of Anthropology, History, and Social Medicine, University of California, San Francisco, 3333 California Street, Suite 485, San Francisco, CA 94143-0850, USA

## Abstract

To understand how older adults perceive and navigate their neighborhoods, we examined the implications of activity in their neighborhoods for their health. We interviewed 38 adults (ages 62–85) who lived in San Francisco or Oakland, California. Seven key themes emerged: (1) people express a wide range of expectations for neighborliness, from “we do not bother each other” to “we have keys to each other's houses”, (2) social distance between “other” people impede a sense of connection, (3) ethnic differences in living arrangements affect activities and activity locations, (4) people try to stay busy, (5) people able to leave their homes do many activities outside their immediate residential neighborhoods, (6) access to a car is a necessity for most, and (7) it is unusual to plan for the future when mobility might become limited. Multiple locations influence older adults' health, including residential neighborhoods. Older adults value mobility, active lives, and social connections.

## 1. Introduction

The phenomenon known as “aging in place” refers to the people wanting to stay in their residence as they age [1–3]; indeed, in the US only, about 5% of people age 55 and over move each year, and half of those who do move stay in the same county [4]. Therefore, understanding the dynamic of older adults in their residential neighborhoods is important for social policy and public health programs in an aging US. As people age and their physical mobility decreases, it is assumed that their geographic world shrinks [5]. While it is relatively unclear at what ages, what levels of functional ability, or in what ways or why older adults pare down the territory in which they act, the residential neighborhood is assumed to be at the center of range. Here, neighborhood refers to individuals' perceptions of their residential environment. This could be a historically recognized area with a name (e.g., Chinatown) or an area that is bounded by certain streets generally accepted by those who live within it to be a neighborhood. In this paper, we examine the perceptions and uses older people make of their neighborhood and the implications for health. 

A review of the quantitative literature (1997–2007) describing how neighborhoods might be associated with health for older adults identified some key limitations: (1) primarily cross-sectional studies, (2) not taking into consideration specific characteristics of older people (e.g., functional capacity and household composition), and (3) few studies which featured ethnic minority study samples [6]. Most of the quantitative literature focuses on a particular segment of the older population and describes the negative effects of living in areas with higher proportions of low-income people, highlighting crime, isolation, and psychological distress [7]. As a result, there remains a gap in the literature about what resources neighborhoods might provide to a more socioeconomically and ethnically diverse population of older adults, barriers to accessing those resources, key features or qualities of neighborhoods that support or maintain older adult health, and whether these qualities differ by ethnicity of the older adult.

Qualitative research has examined the meanings of place for older adults and how they cope with loss (e.g., changes in the social interactions, their ability to engage with place as their capacity declines) as they age [8]. While the literature still lacks studies of ethnic minorities [9], because of its approach, qualitative methods can be more expansive than quantitative literature. Qualitative researchers conceptualize space with a focus on social relations, the power dynamics of those relations, how space is produced and reproduced, and how space contributes to identity formation [10, 11].

We sought to address the gaps in the quantitative literature by embarking on a qualitative interview study with the ultimate objective to translate these qualitative findings into survey methodology to do larger-scale studies. Two conceptual frameworks guided our investigation: social and physical insidedness [12] and environmental press [13]. We describe these briefly here. The geographer, Rowles, developed concepts of connectedness to neighborhoods and other places, specifically for older people. Rowles' term for these concepts was “place attachment.” Place attachment is created through peoples' senses of places' social and physical insidedness. Social insidedness comes from everyday social exchanges over long periods of time resulting in an integration into the social fabric and an overarching identification with a locale. Physical insidedness comes from familiarity and routine within specific settings [12]. Places are redefined in the course of engaging with them. The relative importance of any attribute shifts with varied activities and memories [14]. The concept of place attachment has expanded to include cognitive and emotional bonding as well as behavioral, physical, and social connection to a community [15].

Environmental press is one of the earliest and most comprehensive ecological models of aging [13] and suggests how neighborhood environment influences health. Lawton argued that individuals behave within their environments (“person-environment fit”) and respond to environmental demands (“press”) depending on their abilities to cope with those demands. As people age, they experience physical and social losses, such as losses in vision, mobility, cognitive capacity, and in social support provided by kin and friends. These losses affect their interactions with their environments. Accessing resources is a means of coping. This involves a person's ability to perceive the demands that are present, interpret them as manageable, and act appropriately in response to the demand by strategically deploying the assets they command. Environmental press can be positive, negative, or neutral [13, 16–20].

More recent developments in environmental gerontology have emerged and expanded the place attachment and environmental frameworks, explicitly contributing concepts of behavior, agency, and emphasizing that these are dynamic processes [21]. The Wahl and Oswald Conceptual Framework on P-E Relationships in Later Life highlights autonomy, identity, and well-being, proposing two parallel pathways: (1) experiences (e.g., familiar routines and relationships with neighbors) leading to belonging (e.g., place attachment) and (2) behavior (e.g., moving to change conditions as an adaptation to aging) leading to agency (e.g., altered person-environment fit). Belonging and agency both contribute to well-being. As people age, their level of agency to cope with environmental press may shift. If they are or become over time more physically frail, they may be more confined and vulnerable to negative characteristics (e.g., fewer stores, poor public transit, and lack of friends) [22]. Accompanying the changing dynamic where environment can bring more presses on the individual, one's affective connection to neighborhood may change (e.g., friends move away and feelings of connection or belonging weaken). So, if a person lives in a neighborhood for two or three decades, place attachment may increase over time and then decline with deteriorating cognitive and physical function.

The epidemiologic literature investigating how neighborhoods affect health could benefit from stronger conceptual underpinnings [23, 24]. In this literature, health is often studied in the negative, such as risk for morbidity or mortality. As such, a conceptual framework that highlights the environmental characteristics that provoke adaptation is consistent with that literature that emphasizes negative effects of poor areas such as high crime and inadequate services. The concept of belonging highlights social exchanges, routines, and attachment that develop within settings over time. Investigating belonging promotes understanding positive aspects of health, as in well-being and quality of life. Wahl and Oswald's conceptual framework (2010) highlights the intersections of the individual's behaviors within an environment together with their social experience (e.g., connections or attachment). This perspective can capture older adults' activity and how it relates to their environment, as well as identify positive environmental factors that enable older adults to use their neighborhoods. This conceptual background guided this project, as it collected qualitative data from a multiethnic sample to identify the types of resources that people use in their residential settings to maintain or improve their overall well-being.

## 2. Materials and Methods

### 2.1. Data Collection

Face-to-face interviews took place in participants' homes or a location of the participant's choosing. Interviews were conducted in English or Spanish, lasted between 30 and 180 minutes, were digitally recorded, and transcribed verbatim. After the interview, we asked the respondents to answer a brief demographic survey that included a question on self-rated health. Participants received a $25 gift card as a “thank you.” Study procedures were approved by the University of California, San Francisco (UCSF) Institutional Review Board.

To create an interview guide, we reviewed research literature that documented associations between neighborhood environment and health in older adults. We created a list of overarching topics on the basis of reported associations in the research, the two conceptual frameworks (place attachment and environmental press), and the gaps we located (e.g., not taking into consideration specific characteristics of older adults and defining neighborhood as census or administrative boundaries). Examples of these topics are name of the neighborhood, time in neighborhood, positive or negative characteristics of neighborhood, activities undertaken in the neighborhood, changes in the neighborhood over time, typical activities in a week, and other activities. Within each overarching topic, we generated a set of specific questions to elicit people's accounts of their lived experiences, typical activities (e.g., exercise, food shopping and volunteer activities), the person-environment dynamic, how their activities might contribute to place attachment, and whether the environment created press on the person while engaging in typical activities (e.g., if food shopping was difficult because stores were far away).

 In order to allow participants to describe their experiences in their own words, questions were open-ended with probes as necessary. New questions were added on the basis of the analysis of earlier interviews. The new interview questions were then applied to subsequent interviews. For example, several participants in early interviews were very active and going to a variety of destinations on a regular basis either by driving or through adept use of public transit. In subsequent interviews, we asked about typical activities and how they got to the locations, rather than focus more narrowly on activities in the residential neighborhood.

### 2.2. Sampling

We recruited a purposive sample of older adults from diverse ethnic groups with a range of economic circumstances, aiming for a total of 40 participants. We recruited participants through the organizational contacts of the University of California San Francisco's Center for Aging in Diverse Communities and through professional and personal contacts of one of the authors (IHY).

Eligibility criteria included (a) aged 65 or older, (b) self-identified as White, African American or Black, Asian American, or Latino/Hispanic, (c) lived in Oakland or San Francisco, (d) lived in the same residential neighborhood for at least one year at the time of the interview, and (e) spoke either English or Spanish. Recognizing the population trends discussed above, we wanted to include participants from four ethnic groups in our interview sample, those that are strongly represented in the San Francisco Bay Area. We aimed to split the sample of 40 as evenly as possible across the four ethnic groups; moreover, recognizing that in all included groups women have a longer life expectancy than men, we aimed to interview six women and four men in each of the four ethnic groups.

Excluded were people who lived in predominantly low-income neighborhoods. Excluded neighborhoods were determined on the basis of the research team's prior experience in these areas, Chambers of Commerce data, and representations in the local media. There were two reasons for this exclusion criterion. First, was the necessity to keep the amount of variation among study participants to a manageable level given the desire to target a widely diverse group, on the basis of age, sex, and ethnicity. Second, the decision was made to concentrate the sample selection in order to maximize the chances of exploring positive features of neighborhoods.

### 2.3. Analysis

Two of the authors analyzed transcripts by systematically coding text independently, guided by the interview topics as well as themes that emerged from the data [25]. Analysis took place in several stages. All transcripts were coded by the lead author and another coauthor. Transcripts were read separately and assigned codes that related to the topics and questions in the interview guide as well as other codes that they saw emerging from the data. An initial set of codes was developed. On the basis of this initial coding, a preliminary organization of the codes was constructed, loosely grouped together into larger categories or thematic domains. Subsequent transcripts were coded provoking refinement of codes and thematic domains. Coders met to discuss each transcript and the codes. Any differences of view for any of the coding for transcripts were discussed and resolved until consensus was reached. Discussions during the joint coding meetings also identified new and emergent topics and themes. The coders wrote analytic memos to describe the implications and details of these codes and the larger categories that helped organize the codes [25–27]. All codes were assigned to text blocks using QSR NVivo version 2.0 (QSR International 2006). All authors discussed and concurred with the final list of codes that were applied, the subsequent findings and interpretations of data, and linkages to the conceptual framework.

## 3. Results

Our final sample, diverse in terms of ethnicity and sex, comprised 38 persons ages 62 to 85 (see Table 1). When age data were checked on completion of the interviews, two people were found to be under the age of 65. Their data is included in this report because their health and activity status were very similar to that of participants over 65 years old. They lived in a variety of neighborhoods, ranging from the suburban hills in Oakland to Oakland's Chinatown and, in San Francisco, the well-to-do Pacific Heights area, the Castro (known for its gay community), and the Richmond district (known for Russian and Chinese immigrant communities).

 On the basis of the analytic process described above, we identified seven key themes that fit within the Wahl and Oswald person-environment processes and place attachment framework. For experience or place attachment (belonging), there was a cluster of three themes highlighting social relations and living arrangements: (1) people express a wide range of expectations for neighborliness, from “we don't bother each other” to “we have keys to each other's houses”, (2) perceptions of “social distance” between older people and “other” people (e.g., different ages or race/ethnicities) impede a sense of connection in neighborhoods, and (3) ethnic differences in living arrangements affect activities and activity locations—living with extended family, taking care of grandchildren being more common for Latinas. A second cluster of themes highlighted how behaviors might contribute to agency: (4) people try to stay busy, (5) people able to leave their homes do many activities outside their immediate residential neighborhoods, and (6) access to a car is a necessity for most people. A final theme emerged that indicated that many participants (those who were quite socially and physically active) did not anticipate transitioning into another phase of life should their activity levels decline and their relationship with their home and neighborhood environments shift: (7) it is unusual to plan for the future when mobility might become limited.

**People Express a Range of Expectations for Neighborliness, from “We Don't Bother Each Other” to “We Have Keys to Each Other's Houses.”**

Participants described a range of experiences with their neighbors, from detachment to friendships. Having detached or limited social relations with neighbors was common. In general, they expressed satisfaction with the way things were. At times, people noted that over several years, the turnover in neighbors had created a situation where they were not familiar with their neighbors. In these instances, they also pointed out that the newer neighbors were working age and busy during the day. The differing schedules between the older adults and the working adults meant lower likelihood of running into each other coming and going. One woman (82, Caucasian) did not know many of her neighbors, saying:

*I don't know anybody who lives up here. We did know somebody who lived up here, but they moved away, so I guess we don't know anybody else. I know [name], who lives right behind us. … The other people I just wouldn't recognize if I bumped into them on a street.*

An 83-year-old, African American woman had a conflict with her neighbors regarding parked cars. The neighbors eventually stopped parking their cars in a way that blocked other people from walking on the sidewalk and then the relationship stabilized: “Yeah, and so I enjoy them, because it's like I said, they don't bother me and I don't bother them…” From this woman's perspective, a good relationship with neighbors was a relatively distant one, defined by not being a bother to one another.In a couple of instances, people seemed to indicate that this detachment was not completely consensual. A 62-year-old African American woman who lived in a subsidized senior housing apartment building talked about relations with some of her neighbors in a similar fashion:

Participant: *Another lady down the hall, she passed away, she was very nice lady. … but some of these Black people here, they look at you like you're crazy or somethin'. They don't bother with you. *

Interviewer: *Why do you suppose that is? *

Participant: *They don't want to get involved with you*.

Some participants desired detached relations, because they preferred not knowing so much about their neighbors or being fodder for neighborhood gossip. One woman (69, African American) avoided extended conversations with her neighbor across the street so that he would not talk about her to others or attribute comments to her. In another instance, a participant was resigned not to know her neighbors well. An Asian woman (age 74), when asked about changes in the neighborhood, responded that she did not know of many: “Not really, but maybe some.” She added, “You know, here in America you don't, not too much socializing in this neighborhood,” attributing her lack of knowledge of what was happening in her neighborhood to the lack of socializing within it.Participants also described relationships with neighbors at the other end of the spectrum where they looked out for each other's homes, had keys to each others' houses, and called regularly to check on each other. One man (78, African American) talked about keeping an eye out on a neighbor's home when she leaves for a few days:

*Well, like the lady across the street, she's a widow, she's about eighty-three. Whenever she goes to see one of her daughters she'd let me know, “I'm going to be gone three days, four days.” I watch the house to see if anybody's coming around or what have you.*

This participant indicated that he watched her house even when she was not out of town and made a point to talk to her at least once a week to make sure she was okay. Another participant (84, Caucasian woman) explained that a group of her neighbors have each others' house keys:

Interviewer:* When any of your neighbors go away for trips or anything, do they ever ask you to look in on their place? *

Participant*: Well, yeah. We do that for each other. The corner house, myself and the other house, we all have keys to each others' homes. They have my key, I have their keys. Which makes it nice. Helps. *

African American participants were more likely to mention keeping to themselves or not bothering or being bothered by their neighbors than did other participants. One African American woman (age 69) mentioned that she preferred that there were no sidewalks where she lived, because it deterred people from walking around her neighborhood. Latino participants were more likely to highlight social ties to people they knew through church, rather than those in the neighborhood.

**“Social Distances” with People from “Other Groups”**

Twenty of the 38 participants had lived in their neighborhoods for over twenty years. These people often observed that there had been a lot of change in the composition of the neighborhood population and that they used to know more of their neighbors. A common experience was that the participant would recall that when their children were young, they knew neighbors who also had school-aged children. Over time, households would relocate as children moved out. The newer neighbors might be working aged, away during the day, busy with their own young children, and less available for intermittent neighborhood socializing. Sometimes, the lack of familiarity caused uncertainty or insecurity. People's discomfort was frequently a result of perceived social distance from the neighbors, being far apart in age or of a different ethnicity. A man (70, Latino) commented on young people hanging around in the neighborhood, giving him a feeling of insecurity:

*In the outskirts of our neighborhood there's been more kind of young kids congregating on corners. On the business district there's more young Black and Latino kids, maybe sometimes a White kid, too, but Black and Latino kids, kind of acting rowdy, loud.*

Later, in response to a question of whether there was anything he did not like about his neighborhood, he added:

*What I don't like is the sense that it's become a little more dangerous, you know, in terms of reading about assaults, and seeing kids acting out, you know, on the street. You know, fifteen year olds, acting crazy.*

An older woman (84, Caucasian) who lived near a high school mentioned staying clear of the shopping area near her home when it was lunchtime. She commented:

*We used to have some kids walking around and not going to school and stuff like that. But I think that goes on all over. But the only time you're really kind of bothered with it is if you go up here at lunchtime when they're all out having lunch. But you learn to stay home and avoid it so, that's about it.*

Groups of young people were seen as a threat or a nuisance. There was a sense that older adults and young people belonged to separate groups while occupying the same space. People mentioned a sense of vulnerability in part due to being older with less capacity to defend oneself. Another sort of social distance described by some was with regard to relations with people from ethnic groups different from the participant's. Latinos and African Americans mentioned tensions with Asians. A woman (62, African American) who lived in a subsidized senior housing complex mentioned that her Chinese neighbors greeted her, which she found surprising:

*“Cause you know Chinese people don't… Some of them don't talk to Black people. And that was unusual for them when I was walkin' the hall she spoke to me and talked to me and asked me how I was doin”. And I was just surprised that she would talk to me.*

Another woman (85, Latina) described her Chinese neighbors as not particularly friendly:

*The Europeans and the Latinos are more friendly; the Chinese are … they're not so. Well, there's one nearby to me. The only thing [she/he] says is “hello”, that's all. [She/he] doesn't come by to talk, just a hello, that's all*. [Translated from Spanish]

Language may have been a factor in this dynamic around social distance. Another Latina (age 65) was concerned that storeowners in her neighborhood were taking advantage of the residents by overcharging their merchandise:

*The only problem we have with the stores, is that almost all are run by Arabs. They are, how can I say this, abusive, because they have checkout machines. The items have very small labels and the prices are always faded so it's not possible to see how much something costs. After you pay, they give you a receipt, but there's no ink, so you can't see what the price was. This is a problem for us.* [Translated from Spanish]

Asian respondents were less forthcoming about the topic of social distance though they commented about ethnic composition of their neighborhoods. An 80-year-old Asian man described that the proportions of Chinese neighbors changed over the 50 years he lived in the neighborhood. During some periods, there were more and during other periods, there were less. He mentioned this fluctuation two or three times during the interview. When asked what this fluctuation meant for his experience living in the neighborhood, he would not or could not say. A 74-year-old Asian man said it made no difference to him if his neighbors were Asian or not. Yet, he was clear that some of his closer neighbors were Chinese, while he himself was Filipino. He did say that he liked to go places to engage with Filipinos, “I will always try to go to the place where there are so many Filipinos. To me, it's enough. But when I see Filipinos, I talk to them.”

**Ethnic Differences in Living Arrangements Affect Activities and Activity Locations**

Latina participants tended to live with other family members, in particular adult children, more than did the White, African American, or Asian participants. Four of the Latinas lived with adult children. In two of these instances, their primary activity was to look after grandchildren during the work week. In contrast, one White woman (age 71) lived with her adult daughter. However, it was not until the end of the interview, when asked whether neighbors looked after her home when she went out of town that she mentioned her daughter lived with her. The woman did very little with her daughter regularly, including sharing meals. One African American man (age 66) lived with two of his grandsons who would drive him to places, because he had chronic health problems and some difficulty walking. An Asian couple (both aged 74-years) had retired from work in the Philippines and were living with an adult son. Apart from these four people, all the other African American, White, and Asian participants lived alone (*n* = 14) or with spouses (*n* = 12). Similar to the Latina women, one of the Asian women (age 81) had been living with one of her sons looking after her grandchildren (e.g., taking them to school in the morning, picking them up in the afternoon, and cooking dinner for the family). As the grandchildren grew up and became involved in more activities, she then moved into an apartment in a nearby city that had been purchased for her by her children.

Living arrangements somewhat affected use of services, in combination with an individual's gender and type of neighborhood (i.e., whether there were retail services close by and/or easy access to public transport). Living in close proximity to family was important for all participants. Those who had children living close by could easily get assistance when necessary. For example, an 85-year-old Latina woman was an avid gardener; her son would help her carry large bags of soil or mulch. People who lived alone did all their own food shopping, with the exception of one person who had significant mobility issues. If people lived in a suburban area with no retail close by, they would drive, often selecting the destination on the basis of prices or if they had other errands to do on the way. If people lived with a spouse, usually the woman did more of the food shopping. For one couple who lived close to shopping, the man did all the shopping because the woman had difficulty walking. They had a fixed income. He used the shopping list as his reason for walking in the morning to look at price differences at the various stores close to their home. A Latina woman (age 66) who lived with her daughter and the daughter's family might accompany her daughter to the store; however, most of her activity day during the week was at the senior center near her daughter's work and her granddaughter's school.

**People Try to Stay Busy**

Many of the participants were very active with social activities, work, volunteering, classes, and leisure travel. Several people talked about being “on the go,” wanting to get out every day. In some cases, people intentionally went out to keep mentally and physically active. One woman (69, African American), who was in poor health by her own assessment, commented on how she likes to be on the go:

Interviewer*: Would you say that you spend more time inside at home than out and about? Or is it half and half? *

Participant*: I would say maybe half and half because I'm a goer. Yeah, and if I feel okay, I be out. *

Another woman (68, Latina) talked about being involved in many different activities:

*Well, I don't have family, I don't have anyone. I am not going to sit around. I am involved in many things with the church and other things I do during the day. I don't stay here*. [Translated from Spanish]

Ten of these 38 respondents participated in regular and varied volunteer activities. These included helping drive people who attend the same church to doctor's appointments, holding premature babies in a hospital clinic, visiting patients in hospitals, and helping at a meal program for the homeless. Even people who did not drive but who were interested and had the time did volunteer activities at home. For example, an 80-year-old Asian woman knitted hats and blankets for premature babies in the hospital: “I don't have to go anywhere; I just stay home and do it. And what I do is I knit hats and doilies for the hospital, and make baby afghans.” Activity levels seemed to be a result of personality (being more or less outgoing), physical functioning (ability to get out), and the desire to maintain a similar level of activity as when the person was younger (possibly to retain a younger outlook). One woman (82, Caucasian), when talking about not knowing many of her neighbors, mentioned that she's more introverted and likes to stay at home:

*My husband is just the opposite, he likes people. He thinks I should be more active than I am, but I'm not. I remember that in my family, my mother was the extrovert. She was the one who knew everybody and their business. But I just don't. I'd rather stay home and read.*

A 78-year-old African American widower mentioned forcing himself to get out to socialize and do things:

*Well, if you go to a senior place you're around somebody all of the time, and they have activities going on. So that's the key to me to going someplace else where they have activities going on. … Activities for your body and for your mind.*

A 64-year-old African American man, who was in relatively frail health (with gout and arthritis), regularly took a bus to his former neighborhood in order to spend time with friends. He was living in subsidized housing for seniors, located in a thriving, vibrant neighborhood in San Francisco. His friends live in an area, known for crime and disadvantage, where there is a larger concentration of African American residents. This man may not have had place attachment in his residential neighborhood, but he did have it in another neighborhood, which he regularly visited.

Perhaps in part because people are generally busy in mainstream US culture, older adults express a value in staying busy. There did not seem to be a difference amongst respondents by gender or race/ethnicity about the level of busyness [28, 29].

**People Able to Leave Their Homes Do Many Activities Outside Their Immediate Residential Neighborhoods**

Half of the participants went out of their neighborhoods at least once a week for a variety of reasons. In addition to the activities listed above, weekly activities included going to the movies, participating in hobby or social groups (e.g., bowling league and hiking group), food shopping, caregiving for a friend or relative, window shopping at the mall, or visiting family. Other regularly occurring (monthly or quarterly) events included going to doctor's appointments and picking up prescriptions. The neighborhood was not a key location for the majority of these activities. The most common activity done close to home, mentioned by 13 of the participants, was walking in their neighborhoods. Half of the participants lived close to retail areas with a large array of businesses or were within easy walking distance to smaller commercial districts where there might be a café, a few stores, and a couple of restaurants.

Combined with the emphasis of striving to be busy and on the go described above, this theme highlights the sometimes extensive geographic distance covered by many older adults on a regular basis. While each person's sense of neighborhood physical boundaries differed, activities outside perceived residential boundaries were very common. For example, an 82-year-old man went to the movies twice a week, generally travelling on foot and by bus to go to his favorite theatre about three miles away from his home. A 69-year-old woman drove about seven miles from her home in Oakland to Oakland's Chinatown, two to three times per week, to volunteer activities and buy food. A 78-year-old man travelled by bus Monday through Friday morning five miles to a senior center in the Mission District of San Francisco to visit with friends.

Two people did not leave their neighborhoods. One woman (age 63, African American) had severe arthritis and needed someone to do her grocery shopping for her. Her busyness came in the form of watching television and speaking to siblings on the telephone frequently. The other (an 84-year-old Asian woman) lived in a retail-rich area of San Francisco and did some errands in her neighborhood. When she left the neighborhood, it was for doctors appointments.

**Access to a Car Is a Necessity for Most**

Related to the theme above, having easy access to a car was a perceived to be a necessity. Twenty-one people either drove themselves or had access to a car when, for example, a child or grandchild would drive the person where they needed to go. A woman living in Oakland (72, Caucasian) said, “Well, we wouldn't be able to stay here without being able to drive. I suppose we could use taxis, but that would be the only alternative.” A 69-year-old, African American man in Oakland said, “I would say that there are no stores. And that's one of the major difficulties of living here is that if you don't have an automobile you're up the creek.”

**It Is Unusual to Plan for a Future When Mobility Might Be Limited**

As noted above, we spoke to many people who were socially and physically active, commonly driving outside of their neighborhoods for their activities. We asked people if they had made plans for a time when they might not be so mobile or able to get out so easily (e.g., if they could no longer drive). Five participants were living with an adult child. One couple who had retired in the US from another country, assumed that at a certain point when they were frailer, they would move back to their home country for access to affordable support services. A Latina woman, widowed, originally from Nicaragua, mentioned a similar plan. The others assumed they would continue to live with their adult child and would be able to rely on them. Three participants mentioned that if a time came when they could not get around easily, they would likely move in with an adult child living close by. Some people indicated that they would move to an institution. Others mentioned that they have thought about having a paid caregiver move in to their home. More commonly, people had not given too much thought about it:

*I think I'm just going to rely on my daughters, or my granddaughter by that time. … I have a couple of girlfriends who live up here in the same situation I am, and, you know, we have talked about that, how maybe we can help each other drive or something. But, no, I have to admit I have not given it a lot of serious thought. (69, African American woman)*

Most of the participants adhered to the aspiration to age in place and stay in their current living situations for as long as possible. Given the high level of activity that these older adults maintained and their reliance on the car to be so active, we anticipate that it will become increasingly important to understand how to support older people and their desired activities and lifestyles as they become frailer.

## 4. Discussion

The US population is aging and is increasingly non-white. Current population projections for the US predict that by 2050, the proportion of non-Whites over age 65 will double (from 19% to 39% of the population of age 65 and older people), the proportion of Latinos will triple (from 6% to 18%), the proportion of African Americans will increase by one-third (from 9% to 12%), and the proportion of Asians will nearly triple (from 3% to 8%) [30]. Understanding how neighborhoods and other places affect older adults from different ethnic backgrounds could contribute to policies to address ethnic health disparities.

Through qualitative interviews, we learned about urban older adults' activities both in their residential environments and elsewhere. Participants spent time in their neighborhoods walking and had varying levels of engagement with their neighbors. For participants who were physically able to move about, other than walking or socializing, if the neighborhood did not include retail locations (as was more common in Oakland than San Francisco), then it was not the setting for regular activities. Participants drove their cars to many other destinations to volunteer, exercise, shop, and socialize.

 When applied to these data, the Wahl and Oswald conceptual framework (2010) uncovers some new perspectives on the neighborhood-health dynamic for older adults. Since many of these older adult participants maintain a high level of “busyness” and travel to nonneighborhood locations for a variety of activities, this suggests that it is common to live on a geographic scale greater than the residential neighborhood and that social and material needs are fulfilled by doing activities in a broader space. This has implications for social policy addressing “aging in place,” suggesting the need to provide access to spaces beyond the residential setting. The neighborhood does provide opportunities for social interactions and at times social connections, a basis for the experience-to-belonging piece of the Wahl and Oswald framework (2010). But for the most part, participants in our study largely described detached and distant relations with their neighbors and furthermore expressed satisfaction with this state of affairs. Indeed, the social distance theme reflects a trajectory from that of a form of place attachment (knowing ones neighbors well and having lots in common because children are going to the same schools) to a position of feeling a poorer fit with the neighbors, a form of environmental press, with the change in composition to households that are different in age and/or ethnicity. At the time of the interview, for a relative few, the social ties within neighborhoods were positive characteristics, but in most other cases, the social interactions were sources of tensions or negative environmental press, using the language of Lawton's person-environment framework. Lawton's conceptualization of environment encompassed the personal environment (e.g., spouses and coworkers) and the group environment which referred to the influences of an aggregation of individuals (e.g., neighbors) [31, 32]. The age, race/ethnicity, and language composition of others in the neighborhood contributed to whether these factors were perceived to be part of the press that the environment imposed, and that limited engagement in the neighborhood or, conversely, as a resource residents could used to be meet environmental demands.

We found that people prefer to stay busy and their ability to do so is heavily dependent on having access to a car. Indeed, while all but three people very much wanted to continue living where they were, their primary social and shopping activities occurred outside of their immediate neighborhoods. When asked to think about a time when they might not be able to drive or get around on their own, most people had not given serious consideration as to what they would do under those circumstances. Therefore, for those without access to a car or for those who have no relatives close by and who would likely experience constrained mobility in the future, the features and resources within the neighborhood are and would be important.

As concepts in environmental gerontology have been refined in the last twenty years, the dynamic process of aging has been more explicitly incorporated [22, 33, 34]. With our participants, chronological age did not clearly correspond to physical function or limitations. The youngest three participants, all African American, were in the poorest health. This is consistent with the trend that African Americans develop chronic conditions at younger ages than their White counterparts [35–37]. On the other hand, one of the oldest participants, also African American, had no chronic conditions, and was extremely active, visiting people in the hospital, attending community meetings, and active in her church. Chronological age is not necessarily the most optimal categorization for these participants. These complexities further corroborate the Wahl-Oswald conceptual framework (2010), especially the pathway from agency to identity. Moreover, the arrows along this pathway could potentially also be bidirectional, with the possibility that identity affects behavior, and in turn agency. For our participants, “identity” is tied to group identity, which is informed by social definitions and positions, often less tied to geography, and which motivate behaviors through which individuals seek to affirm and reinforce those identities.

Our longer-term objective in conducting these interviews is to translate the findings to conduct larger-scale survey research. The quantitative research literature on neighborhood-health associations for older adults sometimes uses age (as measured by the proportion of people age 65 and older in the census tract) or ethnic composition (quantified by measures of segregation) among the important demographic characteristics to describe the neighborhood. Our qualitative study supports the significance and continued inclusion of these variables. Our participants confirm what past research, in particular on intentional communities for older adults or age-segregated residential facilities [38, 39], has found, namely, that older people felt more comfortable if there were other older persons living nearby. There was a sense among our participants that younger neighbors were busy with their lives, coming and going, with not much time or interest in older people or any of the other neighbors.

 For ethnic composition, existing evidence and our study findings are more equivocal. Studies have reported that African Americans who live in areas with higher proportions of African Americans have poorer health compared to African Americans who live in areas with lower proportions of African Americans [40, 41]. Other studies have reported that older Latinos who lived in areas with higher proportions of Latinos have better health than Latinos who live in areas with lower proportions of Latinos [42, 43]. The participants in this study did report tensions or uncomfortable interactions with people from other countries or who spoke other languages. Feeling different than, being taken advantage of, or overlooked by racially and ethnically dissimilar neighbors appeared to constitute sources of environmental press with negative effects on neighborhood engagement. Ethnic diversity can be experienced as dissimilarity, as not belonging, as the opposite of place attachment, when residents perceive it to stand in the way of forming social ties to neighbors. The literature also clearly shows that racial/ethnic concentration, when it is a product of segregation, marginalization, and disinvestment in particular communities, is not conducive to health. Our qualitative data support the notion that rather than neighborhood racial/ethnic composition, in and of itself, being important to health, it is a combination of a neighborhood's composition and attendant social, economic, and political resources—or a lack thereof—that are meaningful for residents' health.

 Epidemiologists seek to conduct large-scale, longitudinal studies in multiple locations to produce generalizable population-based findings. The findings from this qualitative research project can be used to inform the contents of a survey for a larger-scale study. On the basis of the key themes reported here, future studies should ask questions about older adults' perceptions of their neighborhood boundaries, where else they regularly spend time, the extent to which they are influenced by the social and physical environment of other neighborhoods in which they spend a significant amount of their time, and their use and reliance on a car or public transit. Future research could extend the Wahl-Oswald framework to these other locations, sometimes referred to as activity spaces [44] and consider the possibility that older adults find and experience place attachment in these more geographically distant places as well as close to home. To date, modest associations have been reported for the neighborhood influence on health status for older adults. Investigating other activity spaces for their resources, demands, and attachments could suggest other mechanisms through which place influences health for older adults.

## Figures and Tables

**Table 1:** Descriptive information of respondents (*n* = 38).

Demographic characteristics	Overall *n* (%)
Age	Mean = 74 (range: 62–85)
Sex	
Men	14 (37%)
Women	24 (63%)
Race/ethnicity	
White	9 (24%)
African American	9 (24%)
Latino	10 (26%)
Asian	10 (26%)
City	
San Francisco	20 (52%)
Oakland	18 (48%)
Housing tenure—own	25 (66%)
Educational attainment	
Did not graduate from high school	6 (16%)
High school graduate	4 (11%)
Some college	10 (26%)
College degree	8 (21%)
Graduate degree	6 (16%)
Missing	4 (11%)
Living arrangements	
Lives alone	18 (47%)
Lives with spouse or significant other	14 (37%)
Lives with adult child	6 (16%)
Car ownership—yes	21 (55%)
Years living in neighborhood	
<10	10 (26%)
11–20	7 (18%)
21–40	13 (34%)
41+	7 (18%)
Self-rated health	
Excellent	4 (11%)
Very good	15 (13%)
Good	9 (24%)
Fair	5 (13%)
Poor	2 (5%)
Missing	3 (8%)
